# EpicPCR 2.0: Technical and Methodological Improvement of a Cutting-Edge Single-Cell Genomic Approach

**DOI:** 10.3390/microorganisms9081649

**Published:** 2021-08-02

**Authors:** Véronica L. Roman, Christophe Merlin, Marko P. J. Virta, Xavier Bellanger

**Affiliations:** 1 Université de Lorraine, CNRS, LCPME, F-54000 Nancy, France; veronica.roman@univ-lorraine.fr (V.L.R.); christophe.merlin@univ-lorraine.fr (C.M.); 2 Department of Microbiology, University of Helsinki, 00790 Helsinki, Finland; marko.virta@helsinki.fi

**Keywords:** PCR-based technic, epicPCR, single-cell metagenomic, taxonomic assignment, high throughput sequencing

## Abstract

EpicPCR (Emulsion, Paired Isolation and Concatenation PCR) is a recent single-cell genomic method based on a fusion-PCR allowing us to link a functional sequence of interest to a 16S rRNA gene fragment and use the mass sequencing of the resulting amplicons for taxonomic assignment of the functional sequence-carrying bacteria. Although it is interesting because it presents the highest efficiency for assigning a bacterial host to a marker, epicPCR remains a complex multistage procedure with technical difficulties that may easily impair the approach depth and quality. Here, we described how to adapt epicPCR to new gene targets and environmental matrices while identifying the natural host range of SXT/R391 integrative and conjugative elements in water microbial communities from the Meurthe River (France). We notably show that adding a supplementary PCR step allowed us to increase the amplicon yield and thus the number of reads obtained after sequencing. A comparison of operational taxonomic unit (OTU) identification approaches when using biological and technical replicates demonstrated that, although OTUs can be validated when obtained from three out of three technical replicates, up to now, results obtained from two or three biological replicates give a similar and even a better confidence level in OTU identification, while allowing us to detect poorly represented SXT/R391 hosts in microbial communities.

## 1. Introduction

The dissemination of antibiotic resistance genes (ARGs) is one of the biggest challenges faced by human and veterinary medicine [1]. ARGs are not homogeneously distributed in bacterial genomes but are rather clustered within mobile genetic elements (MGEs), that ensure their interbacterial mobility [2]. Identifying MGEs bacterial hosts along their dissemination routes is of prime importance to understand and control the spread of ARGs. So far, this question has mostly been addressed by non-targeted approaches, such as shotgun metagenomics, which suffer from several technical limitations such as a lack in coverage depth when it comes to identify bacterial hosts of rare ARGs/MGEs [3,4,5]. Moreover, linking plasmids with their hosts by shotgun metagenomics is inherently difficult because of the discontinuity between replicons. These limitations may be overcome using few recent approaches such as Hi-C (High-throughput chromosomal confirmation capture) [6] or epicPCR (Emulsion, Paired Isolation and Concatenation PCR) [5,7]. EpicPCR relies on a fusion-PCR linking a functional sequence of interest to a 16S rRNA gene fragment from the host. An epicPCR amplification is performed using a template consisting in polyacrylamide beads-entrapped cells dispersed in a stable emulsion to avoid amplifying cross-genome products and, for increasing the desired PCR product quantity, an additional nested-PCR step is carried out during which blocking-primers (BPs) are used to prevent the formation of chimeric amplicons [7]. So far, epicPCR has only been reported for the taxonomic assignment of some ARGs and class 1 integrons that appeared as relatively frequent marker in bacterial communities [8]. In a twin work very recently published elsewhere [9], we explored in depth the environmental host range of another ARG carrying-MGE, namely the integrative and conjugative elements (ICEs) of the SXT/R391 family [10], using different culture-based and molecular-based approaches. Here, we report in detail on significant improvements of the epicPCR workflow made from the original protocol described by Spencer et al. (2016) and Hultman et al. (2018) that allow for pushing forward the limits of the epicPCR. By doing so, we identified several environmentally abundant and never before described SXT/R391 bacterial hosts and proposed a way to qualify these identifications with a system of confidence levels. We also discuss whether the modifications made would be suitable or directly applicable or not in future studies concerning any new target that would be investigated.

## 2. Materials and Methods

### 2.1. Bacterial Strain

The strain CM527 is an *Escherichia coli* MG1656 [11] deriving bacterium containing the SXT_MO10_ element initially isolated from a pathogenic *Vibrio cholerae* strain [12]. *E. coli* MG1656::SXT_MO10_ (CM527) cells were grown in LB broth without antibiotic (160 rpm, 30 °C) for 16 h before cells were used for improving/testing epicPCR conditions.

### 2.2. Sample Collection and Microbial Cells Extraction

Three water and three sediment samples were collected from the Meurthe River (Malzéville, Lorraine, France; 48°43′16.0″ N 6°10′34.2″ E) on September 2019. These choices have been made as the vast majority of SXT/R391-carrying bacteria have been isolated from water or fecal samples and that the sampling point is precisely located in a river downstream the release point of a large wastewater treatment plant (500,000 equivalent inhabitants). Moreover, although the conductivity of river waters usually ranges from 50 to 1500 µS/cm [13], that of the Meurthe River water at the sampling point reaches 4500 µS/cm [9], that may favor the survival/growth of some already known SXT/R391 hosting bacteria as *Vibrio* sp. [14]. Sonication treatments were conducted in attempts to disaggregate cells embedded in water suspended particles or in sediment particles. For water, 45 mL of samples were sonicated for 15 s, 30 s, 45 s, 1 min, 2 min, 5 min or 10 min at 26 W in an ultrasonic bath, just once or twice with a 60 s interval. For sediment samples, 0.5 g or 1.0 g of sediments were dispersed in 700 µL of 1 × PBS using a vortex, and then sonicated two or three times as described for water. 350 µL of sonicated samples were delicately deposited on the top of a 2 mL tube containing 1 mL of a 53.3% Histodenz solution (i.e., 0.8 g·mL^−1^), before being centrifuged at 4 °C for 20 min at 5000× *g*. The top and middle phase were centrifuged again at 4 °C for 10 min at 13,000× *g* after what the supernatant was removed. These steps were performed several times in parallel for each sample, after what the cell pellets obtained from the same sample were pooled and re-suspended in 50 µL of 1 × PBS solution. Water samples were also proceeded without sonication step. In that case, cells were recovered by centrifuging 200 mL of each water sample (40 min at 8000× *g*) before cells were re-suspended in 100–300 µL of molecular biology-grade water. All samples were processed within 1 h after collection and kept at ambient temperature (*ca*. 20 °C) during transportation. When required, recovered cells were mixed with glycerol (20% final concentration) and stored at −80 °C until use.

### 2.3. EpicPCR

#### 2.3.1. Cell Enumeration and Bead Formation

For enumeration, cells aliquots were stained with SYBR Green I (Invitrogen, Waltham, MA, USA), filtered on a track-etched polycarbonate membrane (Whatman Nucleopore, 0.2 µM) and enumerated on an epifluorescence microscope in order to employ the appropriate cell number (i.e., 1–2 × 10^7^ cells) in beads formation, and to ensure the absence of cell aggregates as much as possible. Viability of isolated cells recovered after sonication treatments was determined using the LIVE/DEAD^®^ BacLight™ staining kit and a flow cytometer (BD AccuriTM C6, BD Biosciences, San Jose, CA, USA) equipped with a 50-mW laser emitting at 488 nm, as described previously [15]. The polyacrylamide beads were done as described by Hultman et al. (2018) with the following modifications. Prior bead formation, cells were resuspended in 30 µL of Nuclease-Free Water (Promega, Madison, WI, USA). Then, bead-entrapped cells were stained with SYBR Green I (Invitrogen) and observed to determine the occurrence of beads containing none, one or several cells. According to the Poisson law and considering that there is no cell aggregate, when a sample contains 90% of empty beads, the probability to get beads containing two cells is *p* = 4.5 × 10^−3^. When the frequency of the targeted gene/sequence in the population (*f*) is considered, the probability of having beads containing two cells of which one carries the target is *p* = 2*f* (4.5 × 10^−3^). Assuming this quite low probability whatever the *f* value/target, but also that environmental cells tend to be aggregated to each other, we only considered for further analyzes samples containing more than 90% of empty beads and for which 85% of non-empty beads carry only one cell. In this case, we assume the probability of having two cells in the same polyacrylamide bead, one of which carrying the targeted gene, can be considered as negligible.

#### 2.3.2. Fusion, Nested and Blocking PCRs and Sequencing

As described by Hultman et al. (2018) with few modifications, fusion PCR amplifications (epicPCR first DNA amplifications) were performed using cells entrapped in polyacrylamide beads as template. Briefly, these PCRs were done using Phusion DNA Polymerase (NEB) with either the GC or HF buffer, and an annealing temperature of 51 °C. The resulting amplification products were extracted/purified using the Monarch PCR & DNA Cleanup kit (NEB), eluted in 30 µL of Nuclease-Free Water (Promega), and stored at −20 °C. Undiluted or a 10-fold dilution of the fusion PCR products were used as template DNA in either (i) nested PCR amplifications where blocking primers (BPs) were added as originally described by Spencer et al. (2016), in order to prevent random extension of unfused PCR products or, (ii) in “blocking PCRs”. Blocking PCRs have been inspired by the nested PCRs used by Spencer et al. (2016) but they are reactions in which BPs are the only primers used in order to get a full blocking of undesired fusion PCR products before carrying out a subsequent nested PCR. All the nested PCRs were performed with BPs as described by Hultman et al. (2018) with one of the four following BP concentrations: 3.2 µM, 0.32 µM, 0.032 µM or 0 µM (as a control). For the blocking PCRs, the mix used was the same as the one for the nested PCRs, excepting the absence of nested primers. The amplification program of blocking PCRs was the same that the one of nested PCRs depicted by Hultman et al. (2018). Nested PCR products were purified using the Monarch PCR and DNA Cleanup kit (NEB) and sequenced on the Illumina MiSeq 2 × 250 platform (Genewiz, South Plainfield, NJ, USA). The primers used in this study are listed in Supplementary Table S1.

### 2.4. 16S rRNA Gene Amplification and Sequencing

The PCR amplifications of the V3-V4 region of the 16S rRNA genes were done on total microbial DNA with the 515F and 806R primers (Table S1) using Phusion polymerase and HF buffer (New England Biolabs, Ipswich, MA, USA). The cycling conditions were as follows: initial denaturation at 98 °C, followed by 28 cycles at 98 °C for 10 s, 58 °C for 30 s and 72 °C for 15 s, and a final extension for 5 min at 72 °C. The PCR products were pooled, cleaned (Monarch PCR & DNA Cleanup Kit; New England Biolabs, Ipswich, MA, USA) and sequenced on Illumina Miseq (2 × 250) (Genewiz, South Plainfield, NJ, USA).

### 2.5. Sequencing Data Analysis

EpicPCR sequence analyses were mostly performed as described by Hultman et al. (2018). Briefly, reads were joined using Pear [16] with default options, and sequence quality was checked with FastQC [17]. Primers and short reads (<250 bp) were removed before sequences were split into 16S rRNA and SXT/R391 sequences using cutadapt [18]. Unique sequences were identified using USEARCH -fastx_uniques command [19]. OTUs were clustered and reads were mapped to reference sequences with the USEARCH -cluster_otus command with -minsize 2 parameter. Taxonomic classification of OTUs was done using the USEARCH -sintax command, using the ltp_16s_v123 database with the classifier cutoff = 60. A list of recovered OTUs is given in Supplementary Table S2. OTUs were considered for further analysis and validation when composed of at least 10 reads and detected in either technical or biological duplicates. Statistical analyses were performed using R 3.4.1 [20].

For the standard 16S rDNA metagenomics, reads were joined with Pear [16] using default options, sequence quality was checked with FastQC [17] and primer sequences and short reads (<330 bp) were removed using cutadapt [18]. Unique sequences were identified using USEARCH -fastx_uniques command [19]. OTUs were clustered and reads were mapped to reference sequences with the USEARCH -cluster_otus command with the -minsize 2 parameter. Taxonomic classification of OTUs was done using the USEARCH -sintax command, using the ltp_16s_v123 database with the classifier cutoff set at 60. A representative sequence from each bacterial family (constituted of at least 10 reads) was aligned with MUSCLE [21], and a phylogenetic tree was built with FastTree [22] and visualized in iTol [23].

## 3. Results and Discussion

### 3.1. Elaboration of Polyacrylamide Beads Carrying Isolated Environmental Cells

Isolating single environmental cells in polyacrylamide beads is mandatory for the good achievement of epicPCR in order to avoid chimeric PCR fusion products between markers originating from distinct bacteria. Here, we describe how we proceeded for creating beads from the water and sediment samples we collected. If this way to proceed could likely be used with other samples presenting the same characteristics and that some of the modifications we proposed to the protocol described by Hultman et al. (2018), such as those presented in Section 2.3.1, then it is likely to be suitable for all new samples that would be investigated; we do not think the approach presented below would be universal. We therefore want here to insist that, from our point of view, the best way to create polyacrylamide beads containing isolated bacteria for epicPCR purposes should have to be re-evaluated at the beginning of each epicPCR-based investigation. In our first series of experiments, polyacrylamide beads containing bacteria were created using microbial communities from water and sediments sampled in the Meurthe River (Malzéville City, France) in September 2019. Before forming polyacrylamide beads, several sonication conditions were tested, varying the duration and the intensity of the treatments, in attempts to disaggregate cells embedded in water suspended particles or sediment particles. The sonication conditions were adapted in order to reach the maximum effect without detectable loss of cell viability as seen by staining cells with the LIVE/DEAD^®^ BacLight™ kit. Unfortunately, numerous particles of sonicated samples, especially from sediments, had size range and fluorescence intensity range comparable to stained cells (data not shown), thus impairing the proper evaluation of cell viability. Such difficulties when using SYBR Green I for staining cells isolated from eutrophic environments are known for a long [24,25] and can result from autofluorescence of minerals or organic matter. In addition, whatever the conditions used, the sonication step allowed disaggregating particles in smaller ones. Although a 35 µm-mesh cell sieve is used to remove aggregates embedded in polyacrylamide after bead polymerization, in a large majority of cases, the use of sonicated samples for bead formation led to the recovery of a higher proportion of beads carrying small aggregates of few cells compared to non-sonicated samples. Considering the average diameter of bacteria around 0.2–2 µm, obtaining beads carrying more than one cell when disaggregating soil or water suspended particles can be expected. Regarding sediment samples, intrinsically highly charged in cell aggregates, isolated cells were tentatively extracted after sonication using an Histodenz density gradient, but the yield obtained never allowed reaching the minimum amount of 1.10^7^ cells needed to properly create polyacrylamide beads. By taking these constraints into account, and while improving or experiencing other methods to extract a higher cell concentration from sediments but also from biofilm samples, this study was conducted focusing on water samples only. To our hand, it appeared that the more appropriate and time saving option for recovering raw water cells dedicated being embedded in polyacrylamide beads was to vigorously vortex the samples, without any sonication step, before collecting cells by centrifugation. Practically speaking, the adequate volume of water sample corresponding to the 1–2 × 10^7^ needed cells were vortexed for 2 min 30 in a microtube or a conical tube at maximal speed using a Vortex-Genie^®^ 2 mixer, before to be centrifuged at 12,000× *g* for 1 min. The quality of all bead batches used for epicPCR amplifications were analyzed by microscopy as described in Section 2.3.1.

### 3.2. Checking epicPCR Design on Pure Culture

**Figure 1:**
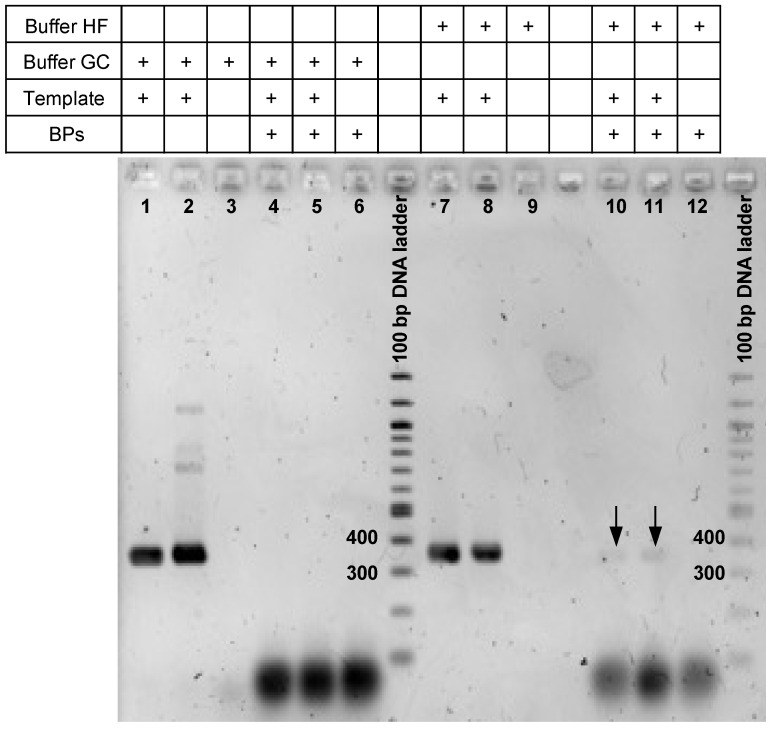
Control epicPCR amplifications targeting SXT/R391 ICEs performed on beads carrying *E. coli* MG1656::SXT_MO10_ as template. Each experiment was done in duplicates with a no-template DNA control, using either the Phusion DNA Polymerase GC or HF buffers. In the nested-PCR step, the use of blocking primers (BPs) has been done as depicted in Spencer et al. (2016), and usually performed so far. All these conditions are summarized in the table upper the gel. The expected size of the desired DNA fragment is 367 bp. Black arrows indicate epicPCR products obtained after performing fusion and nested PCRs using HF but not GC buffer.

The primers used to target SXT/R391 ICEs have been recently designed to target a conserved gene of these elements, traB, by quantitative PCR [9]. Using these primers allows to get robust qPCR amplifications (efficiency: 97.3%; R^2^: 0.999; Quantification limit = Detection limit: 3 copies). Moreover, the sequencing of amplicons obtained with these primers while using environment genomic DNA as template confirmed they only amplified a sequence specific to SXT/R391 ICEs [9]. Therefore, we considered their design to be very good and very likely suitable for being used in epicPCR amplifications. This probable suitable design of the primers for fusion PCRs and nested PCRs as well as blocking reactions targeting SXT/R391 ICEs, was checked on the single strain *E. coli* MG1656::SXT_MO10_. A suspension containing around 1 × 10^7^ *E. coli* MG1656::SXT_MO10_ cells in water was used in order to prepare polyacrylamide beads carrying isolated cells as described by Hultman et al. (2018). These beads were thereafter used for optimizing the settings of epicPCR reactions targeting SXT/R391 elements. To do so, the annealing temperature was first adjusted to 51 °C according to the primer sequence properties (Supplementary Table S1). The fusion PCR and the subsequent nested PCR amplifications were then performed using the conditions depicted in Spencer et al. (2016), i.e., using the Phusion DNA Polymerase GC buffer, but no PCR product resulting from the fusion of SXT/R391 ICE and 16S rDNA gene fragments could be observed in agarose gel electrophoresis, unless BP were omitted (Figure 1, wells #1 and 2 vs. 4 and 5). This difficult amplification in epicPCR was surprising in regards of the amplification robustness that these primers allow to reach in qPCR [9]. We therefore rather considered replacing the GC buffer by the standard Phusion DNA Polymerase HF buffer because its use allows a lower error rate while incorporating nucleotides. This changing indeed enhanced the final amplicon yield but this one was still not enough to get nested PCR product in sufficient concentration for sequencing (Figure 1, lines #10 and 11). During this experiment, the lack of nested PCR products appeared to be linked to the use of the BPs that were otherwise supposed to prevent random extension of unfused PCR products. Considering the primer design was correct, we hypothesized that, at 3.2 µM, BPs prevent the proper nested PCR to proceed by competing with the complementary strand for annealing. Thus, the PCR fusion design has appeared robust, but the nested PCR inhibition and the weak final amplicon yield inherent to the original method when using the BPs were clearly redhibitory for a next NGS sequencing step. However, as described in Section 3.3, this aspect could be significantly improved with a better and careful usage of BPs during the epicPCR process. Regardless, we definitively think such a validation step of the epicPCR design on pure culture would be interesting to be performed with every new target that would be investigated, especially for adapting the PCR parameters (buffer, annealing temperature…).

### 3.3. Tuning the Use of BP to Maximize epicPCR Efficiency on Environmental Cells

**Figure 2:**
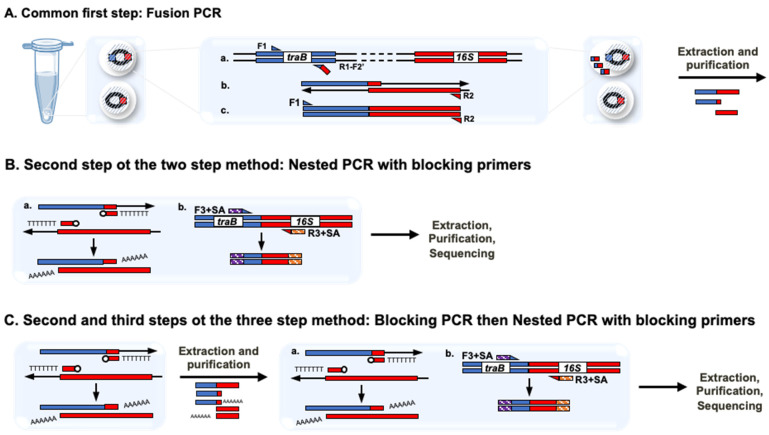
Principle of the two- and three-steps epicPCR protocols implemented. (**A**) Fusion PCR in polyacrylamide beads. (a) Amplification of the traB gene fragment using F1 and R1-F2′ primers. The latter is added at limited concentration and will serve as forward primer for (b) the 16S rDNA gene fragment amplification. (c) Finally, F1 and R2 primers amplify the fused product. (**B**) Nested PCR with blocking primers. (a) Blocking of unfused fusion PCR products using BPs at 3.2 µM, along with (b) a nested PCR on fused products using F3 + SA and R3 + SA primers carrying sequencing adaptors (SA). (**C**) Blocking PCR then Nested PCR with blocking primers. Unfused products were first blocked using only BPs at 3.2 µM, before performing a PCR where (a) potentially residual unfused products were blocked using blocking primers at 0.32 µM, along with (b) a nested PCR on fused products using F3 + SA and R3 + SA primers.

**Figure 3:**
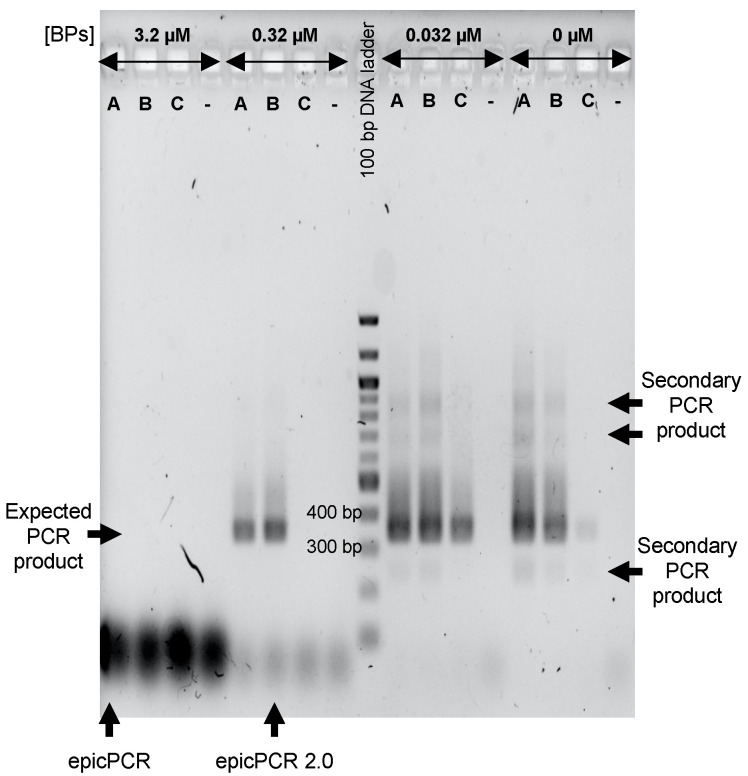
Efficiencies of two- and three-steps epicPCR protocols using different blocking primers (BPs) concentrations. The epicPCRs were run on SXT/R391 carrying bacteria from the Meurthe River water. A and C indicate wells with amplification products from two-steps epicPCR protocols (fusion-PCR on polyacrylamide beads + nested PCR). In the A lane, fusion-PCR products from the first step were used as template DNA in the second step without dilution whereas these products were diluted ten times in line C to circumvent the possible presence of PCR inhibitors. B indicates wells loaded with amplification products resulting from a three-steps epicPCR protocol (fusion PCR on polyacrylamide beads + blocking PCR with BPs as sole primers + nested PCR). The expected size of the final nested-PCR product is around 350 bp (depending on the 16S rRNA gene fragment polymorphism). The conditions of epicPCR as used in Hultman et al. (2018) and that we determined to be the best in our conditions (epicPCR 2.0) are indicated by black arrows. The minus symbols indicate negative controls with epicPCRs run without polyacrylamide beads-template.

In a next step, polyacrylamide beads carrying cells from the Meurthe River water were used to address the BP-associated inhibitory effects as reported in Figure 1. Two approaches were considered. In the first one (two steps protocol), the BP concentrations were modulated during the nested PCR whereas, in the second one (three steps protocol), a set of independent “blocking cycles” consisting of 30 extension reactions was added between the fusion and the nested PCR (Figure 2). The idea behind the addition of these independent “blocking cycles” was to allow the BPs to prevent the possibility of forming chimeric amplicons as before, without impairing the subsequent nested PCR by limiting the BPs side effect. All in all, 3 BP concentrations, 3.2, 0.32 and 0.032 µM, were tested in the nested PCR step whatever the protocol used (two steps or three steps), while BPs were used at 3.2 µM in the blocking cycles of the three steps protocol. The results obtained are presented Figure 3. Here again, the use of the two-steps protocol initially published did not allow getting DNA fragments at the expected size (Figure 3, “epicPCR” arrow). Nevertheless, reducing the concentration of BPs at 0.32 or 0.032 µM, in either two- or three-steps protocols, significantly improved the detection of the expected DNA fragments. In the light of these experiments, we propose a new epicPCR protocol permitting the most efficient blocking reaction together with the best amplicon yield, i.e., the three-steps protocol consisting in a fusion PCR, a blocking PCR with BPs at 3.2 µM followed by a nested PCR run in presence of BPs at 0.32 µM (Figure 3, “epicPCR 2.0” arrow). Here again, we think this modification proposition would be suitable for every new target that would be investigated.

### 3.4. Exploring the Way to Validate OTUs in epicPCR Experiments

EpicPCR has been developed for the taxonomic assignment of moderately rare functional genes. However, the quite low abundances of SXT/R391 element-carrying bacteria in the environment, ranging from 10^−3^ to 10^−7^ SXT/R391 ICEs/16S rDNA copies [9], led us to reach the limits of this approach. In that respect, we focused our study on the Meurthe River ecosystem as it precisely appeared to be quite rich in SXT/R391 ICEs with relative abundances up to 10^−3^ MGEs/16S rDNA copies [9]. Considering (i) that the prevalence of the SXT/R391 ICEs in river communities could easily be as low as 10^−6^–10^−7^ MGEs/16S rRNA gene [9], and (ii) that a single epicPCR experiment hardly involve more than 3 × 10^6^ polyacrylamide beads entrapped-cells, we hypothesized that at only very few cells carrying SXT/R391 elements can be implicated in an epicPCR reaction. In other words, this makes the number of reads, and therefore detectable operational taxonomic units (OTUs), very low, with the risk of missing interesting results if the parameters used for defining OTUs are too stringent. In such case, we had to reevaluate and properly adapt the way usually used to validate each OTU, which means here to identify a bacterial taxon hosting the targeted gene/MGE. In any case, it would be wise and time saving to systematically assess the relative abundance (copies/16S rDNA copies) of the target gene/sequence by qPCR in bacterial populations before each epicPCR experiment for avoiding the study of samples with too rare presence of bacterial hosts of this target gene/sequence of interest.

**Figure 4:**
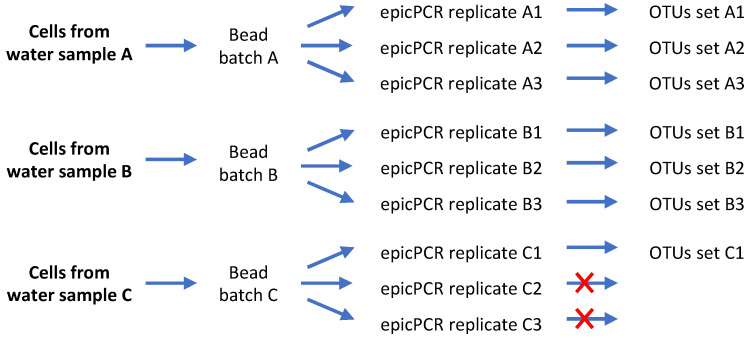
Replicate strategies for taxonomic assignment of SXT/R391 elements in the Meurthe River water by epicPCR. Each bead batch allowed performing the original epicPCR approach using a set of technical replicates that can be considered and analyzed as either triplicates (1 combination) or duplicates (3 combinations if ignoring one of the repeats without statistical a priori). The use of biological replicates implies using OTUs obtained from different bead batches. By doing so, 15 biological duplicates (3 × 3 + 3 × 1 + 3 × 1 = 15 combinations) or 9 biological triplicates (3 × 3 × 1 = 9 combinations) can be considered. Red crosses indicate epicPCR amplifications for which the sequencing of the resulting amplicons did not reach the minimal quality requirement.

**Figure 5:**
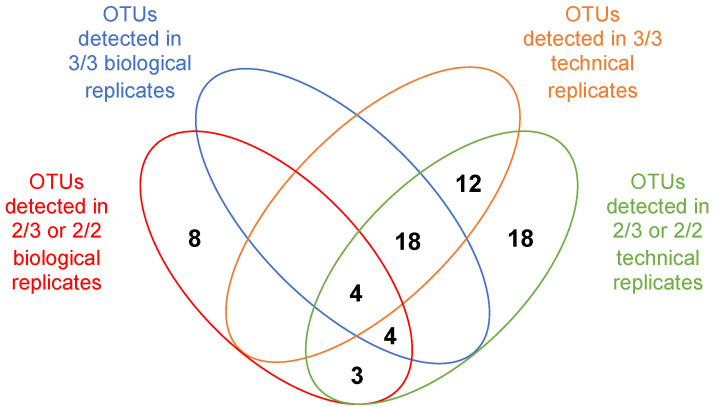
Comparing the influence of the epicPCR replicate strategies on OTU detection. This Venn diagram presents the distribution of the 67 validated OTUs resulting from the taxonomic assignment of SXT/R391 elements in bacteria from the Meurthe River water according to the methodologies used and presented Figure 4 and Supplementary Table S3. The bias toward detecting more OTUs in technical replicates is revealed by the high OTU numbers in the corresponding ellipses.

**Figure 6:**
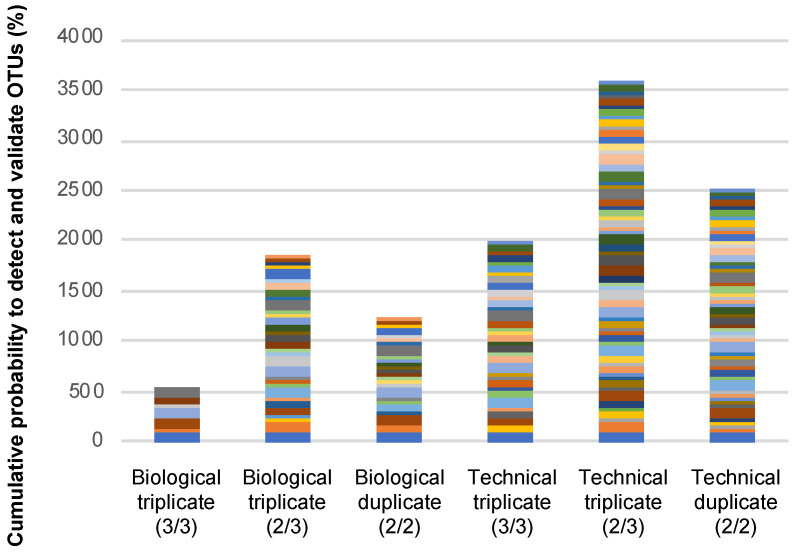
Efficiency of the epicPCR experiment expressed as the cumulative probability to detect OTUs of SXT/R391 hosts from the Meurthe River water. This figure considers each of the 67 validated OTUs mentioned in Figure 5 and Supplementary Tables S2 and S3 using either technical of biological replicates and, according to experimental plan presented in Figure 4. The ratios in brackets indicate the occurrence a given OTU must have between the considered replicates for being validated. Each color/band represents a given OTU.

In the original epicPCR setup [7,8], an OTU was validated when obtained in each of the three technical replicates carried out using a same batch of polyacrylamide beads. Such methodology, now on referred as a “3/3 technical replicates” OTU validation method, was initially proposed to address the possibility of more than one cell becoming embedded in the same bead with only one hosting the target sequence since it would be unlikely to happen twice. However, such validation parameters may appear (i) inappropriate when the target sequence is too rare as its presence in a sub-batch of beads will appear stochastic and (ii) not representative enough of the sample content. To go forward, we considered OTUs obtained from biological replicates, i.e., in epicPCRs performed using different beads batches. The use of biological replicates also makes unlikely the possibility to run twice an epicPCR on a same kind of undesired beads carrying the same two bacterial species. We therefore assumed that the detection of rare OTUs from biological replicates will be at least as robust as the one resulting from technical replicates. This alternative was evaluated by comparing outcomes obtained using the original “3/3 technical replicates” method, to those obtained using “3/3 biological replicates” and “2/3” or “2/2” biological or technical replicates (see Figure 4 for epicPCR replicate strategy). To do so, we determined the significance of detecting a given OTU in all (“3/3” or “2/2”) or most (“2/3”) of the technical and/or biological replicates (Figure 5 and Figure 6). Figure 5 depicts the influence of using a replicate strategy on the number and nature of OTUs that can be detected, while Figure 6 presents the global efficiency of each replicate strategy, i.e., the probability of detecting the maximal number of SXT/R391 hosts, expressed as the cumulative probabilities to detected the OTUs presented in Figure 5.

Of the 128 OTUs detected, 67 were validated as present in at least two replicates (Supplementary Tables S2 and S3). When comparing the way to proceed with duplicates or triplicates, we determined that validating OTUs from technical triplicates is not independent from validating OTUs from technical duplicates (McNemar test, *p* = 2 × 10^−6^), because the considered replicates were performed using the same bead batch (Supplementary Table S3, Figure 5 and Figure 6).

In other words, detecting an OTU in one of the technical replicates is influenced by the presence of the same OTU in the other replicate(s), thus affecting the final set of validated OTUs. Such a dependence relationship has not been evidenced when biological duplicates and triplicates were performed (McNemar test, *p* = 0.27), meaning that the detected OTUs from a biological replicate does not influence OTUs detection in another replicate. At the same time, the biological replicates approach leads to the validation of fewer OTUs than with the technical replicates approach. On principle, the greater OTUs number obtained and the dependence relationship pinpointed when using technical replicates may result from using beads batches having some non-single cells-containing beads. Nevertheless, considering on the rigorous quality controls we implemented and on the rareness of the targeted genes/elements in the studied bacterial populations, we rather favored the idea that all bead batches created using bacteria originating from the Meurthe River water were probably not enough representative of the gene-of-interest carriers due to the rareness and the non-homogenous distribution of the target sequence in the population. By using different bead batches in biological replicates, we assume that we may not detect the rarer SXT/R391-carrying bacteria that would be present in one bead sample but not in the others, and that could be detected when following the technical replicates approach, i.e., when performing technical replicates using a sample presenting an unexpectedly rich proportion of rare SXT/R391-carrying bacteria. Therefore, even if using the “2/3 biological” and the “3/3 technical” approaches looked similar in terms of probability to detect a maximum number of OTUs (Student’s *t* test, *p* = 0.68) (Figure 6, Supplementary Table S2), the biological replicates approach offers a better confidence level for the detected OTUs, but it may not allow detecting the rarer SXT/R391-carrying bacteria in the population. Finally, validating OTUs in “2/3” or “2/2” replicates (technical and biological) allowed detecting the same OTU patterns, but the probability of detecting a given OTU is higher with “2/3” replicate approaches (Table S2, Figure 4 and Figure 6).

All in all, with this comparative work, the epicPCR replicate strategy could be ranked with a decreasing level of confidence (LC) as follow: LC5: “3/3 biological” > LC4: “2/3 biological” = “2/2 biological” > LC3: “3/3 technical” > LC2: “2/3 technical” = “2/2 technical”. The lower level of confidence (LC1) corresponds to the 61 of the 128 detected OTUs that have been found in only one replicate, and therefore that likely cannot be validated with an acceptable risk (Supplementary Tables S2 and S3). We recommend these OTU validation methods and corresponding CLs to be used in all future epicPCR-based analyses that will be undertaken.

### 3.5. Plotting epicPCR Results on a Gold-Standard 16S-rDNA Tree

**Figure 7:**
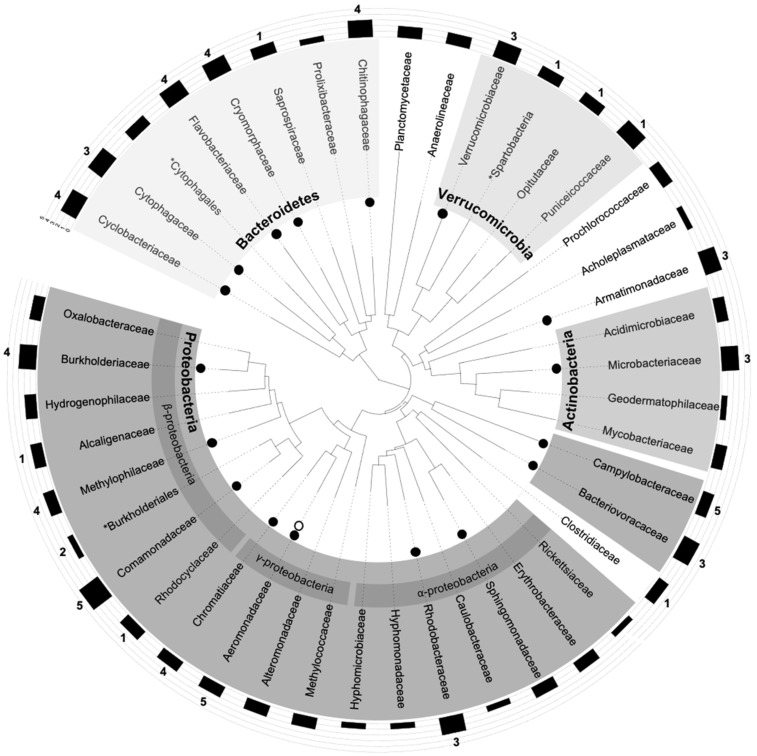
Host range of SXT/R391 ICEs in the Meurthe River water identified by epicPCR. The phylogenetic tree was constructed from family-level OTUs based on 16S rRNA gene amplicon sequences. The confidence levels of the presence of SXT/R391 elements in a bacteria family are indicated by numbers. Open and full black circles indicate taxonomic families where SXT/R391 elements have been previously detected or detected in the present work with a high level of confidence (LC ≥ 3), respectively. The black rectangles display the relative abundances of 16S rDNA reads for the considered family (Log10 (sum of OTU reads from a family/total reads of a sample) × 100,000). Asterisks are for unclassified families and undetermined OTUs are not shown.

Whatever their level of confidence, OTUs identified thanks to the epicPCR approach may appear as speculative until they are compared to/pointed on a gold-standard phylogenetic tree resulting from a metagenomic analysis of the bacterial population also used in the epicPCR amplifications. Here, the total genomic DNA of the bacterial population used to prepare the polyacrylamide beads were extracted before the V3–V4 variable regions of the 16S-rRNA gene were PCR amplified and sequenced. Then, a phylogenetic tree was constructed from family-level OTUs following a gold-standard approach and OTUs of SXT/R391 hosts identified by epicPCR were pointed on the obtained tree (Figure 7). Among the 67 validated OTUs in epicPCR, 17 were assigned to an unknown bacterial family and were not further considered. The 50 remaining OTUs belong to 22 bacterial families, with Aeromonadaceae, Chitinophagaceae and Flavobacteriaceae being the more represented ones clustering 4, 4 and 10 OTUs, respectively. Sixteen of these 22 bacterial families were indeed present in the phylogenetic tree (Figure 7) and, some bacteria belonging to one of those 16 families, identified with the best CL of 5, Aeromonadaceae, have already been described as hosting an SXT/R391 ICE [10]. Bacteria from the Aeromonas genus (Table S2, OTU12), and therefore belonging to Aeromonadaceae, have notably already been recently identified by our group as SXT/R391 hosts in the Meurthe River ecosystem by another approach [9]. These finding strongly suggest an at least partial validation of the obtained epicPCR results. The six bacterial families validated in epicPCR with a LC ≥ 2 but that have not been able to be pointed on the phylogenetic tree were those of the Enterobacteriaceae (LC = 4), Planococcaceae (LC = 4), Shewanellacea (LC = 4), Bacteroidaceae (LC = 2) and, Pseudomonadaceae (LC = 2). Nevertheless, members of Enterobacteriaceae and Shewanellacea isolated in culture-based analyses have already been reported as being SXT/R391 hosts. The inability of pointing an epicPCR OTU in the phylogenetic tree thus appears to not necessarily be synonymous of having identified a false host family of the target gene/element. In the same way, the 61 OTUs detected in epicPCR with the lower level of confidence (LC = 1) (Table S2) can be clustered in 14 bacterial families of which 2 (Idiomarinaceae, Oceanospirrillaceae) are already known to contain SXT/R391 hosts. As for the absence of Enterobacteriaceae and Shewanellacea in the phylogenetic tree, this last finding strongly suggests that epicPCR, especially the epicPCR 2.0 approach, is a powerful technic that can highlight weakly abundant hosts of the targeted gene/element in the population that other metagenomic approaches could likely hardly identified. Considering the obtained results by the side of the SXT/R391 ICEs, our findings demonstrate that the environmental reservoir of these ICEs in the Meurthe River water is mainly composed by bacterial species not identified so far as SXT/R391 hosts, and that this reservoir do not seem to reflect the range of SXT/R391 hosts that the culture-based approaches up to now implemented, mainly in a clinical context, would lead us to suggest.

### 3.6. Synthetic Comparison of epicPCR and epicPCR 2.0 Methodologies

The success of the epicPCR approach, especially for the taxonomic assignment of rare targets, is strongly linked to the efficiency in achieving its multiple steps from the molecular part to the subsequent bioinformatic analyses. In this last section, all the differences and improvements compared to the initial method that we implemented in the present work have been summed up for a better overview in Table 1. Whatever the methodologies used and even if new improvements would be made to the modified methodology we propose, some limitations will very likely always remain in epicPCR approaches, such as the difficulties to be sure of the presence of already know targeted gene/sequence-carrying bacteria in the investigated samples to fully validate the results. One possibility we did not initially consider could be to spike the sample to be studied with few cells of a genetically engineered bacterium in which a unique and artificial allele of the targeted gene/sequence would be inserted. For example, we can imagine, in a study focusing on a housekeeping gene, to clone a fragment of the target containing multiple non-sense mutations, that are unlikely to occur in the environment and easily recognizable during the analyses of the sequencing reads. Identifying such an artificial target in the sole host used to spike the studied samples should definitively validate the quality of the bacteria containing-polyacrylamide beads created and still reinforce the validation of the final sequencing results. Spiking different dilutions/amounts of a genetically engineered bacterium would also advise about the detection limit of the approach in the considered samples. Finally, it should be stressed that the newly proposed epicPCR 2.0 approach does not significantly require more experimental nor analytical time than the initial protocol, while allowing us to get deeper and better characterized results.

## 4. Conclusions

EpicPCR is one of the only techniques available for the taxonomic assignment of relatively rare functional markers in communities, but it remains relatively tricky to implement. With this work, we propose significant improvement of the epicPCR workflow at two main critical levels without burdening the experiments to be done. First, we propose a new workflow to adapt epicPCR conditions, notably by tuning the use of BPs in a blocking cycle that notably increase the amplification yield of the final epicPCR product. Second, we propose an alternative replicate strategy allowing us to increase the level of confidence of the validated OTUs while detecting poorly represented hosts of the marker in microbial communities.

## Figures and Tables

**Table 1:** Comparison of the main characteristics of the initial epicPCR and the improved epicPCR 2.0 approaches.

Characteristics	epicPCR	epicPCR 2.0
Polyacrylamide beads formation	Done once per sample	Done three times in parallel per samples
Beads formation quality check (by microscopy after cell staining)	More than 90% of beads are empty	More than 90% of beads are empty and 85% of non-empty beads only carry one cell
Replicate strategy for performing the fusion-PCR	Triplicated amplification using a single beads batch per sample as template (=technical triplicates)	Single non-replicated amplification performed on each of the 3 beads batches (=biological triplicates)
Fusion-PCR ^1,2^	Performed with primers F1, R1-F2′ and R2 used at 1, 0.01 and 1 µM, respectively	
Step between the fusion- and the nested-PCRs	None	Additional “blocking PCR” step using the blocking primers at 3.2 µM alone
Nested-PCR^2^	Performed with the primers F3 + SA and R3 + SA at 0.3 µM and blocking primers at 3.2 µM	Performed with the primers F3 + SA and R3 + SA at 0.3 µM and blocking primers at 0.32 µM
Minimum of reads for creating an OTU in each replicate	100	10
Preferential OTU validation method	OTU detected in each of the 3 technical replicates	OTU detected in each of the 3 biological replicates
Confidence levels for characterizing OTU validation	None	Yes; from 5, the best one, to 1

^1^ Sole main step shared by the epicPCR and epicPCR 2.0 approaches. ^2^ Primers are named according to Figure 2.

## Data Availability

All 16S rRNA gene sequences have been deposited in GenBank (BioProject accession no. PRJNA661996 and PRJNA662024).

## Supplementary Materials

The following are available online at https://www.mdpi.com/article/10.3390/microorganisms9081649/s1, Table S1: List of the primers used in this study, Table S2: List of OTUs detected in the 3 Meurthe River water samples when targeting SXT/R391 carrying bacteria, Table S3: OTU detection according to the epicPCR methodology as presented Figure 4.

## References

[1] Collignon P., Beggs J.J., Walsh T.R., Gandra S., Laxminarayan R. (2018). Anthropological and socioeconomic factors contributing to global antimicrobial resistance: A univariate and multivariable analysis. Lancet Planet. Health.

[2] Partridge S.R., Kwong S.M., Firth N., Jensen S.O. (2018). Mobile genetic elements associated with antimicrobial resistance. Clin. Microbiol. Rev..

[3] Bengtsson-Palme J., Larsson D.J., Kristiansson E. (2017). Using metagenomics to investigate human and environmental resistomes. J. Antimicrob. Chemother..

[4] Guo J., Li J., Chen H., Bond P.L., Yuan Z. (2017). Metagenomic analysis reveals wastewater treatment plants as hotspots of antibiotic resistance genes and mobile genetic elements. Water Res..

[5] Larsson D.J., Andremont A., Bengtsson-Palme J., Brandt K.K., de Roda Husman A.M., Fagerstedt P., Kvint K., Laxminarayan R., Manaia C.M., Nielsenn K.M. (2018). Critical knowledge gaps and research needs related to the environmental dimensions of antibiotic resistance. Environ. Int..

[6] Stalder T., Press M.O., Sullivan S., Liachko I., Top E.M. (2019). Linking the resistome and plasmidome to the microbiome. ISME J..

[7] Spencer S.J., Tamminen M.V., Preheim S.P., Guo M.T., Briggs A.W., Brito I.L., Weitz D.A., Pitkänen L.K., Vigneault F., Virta M.P.J. (2016). Massively parallel sequencing of single cells by epicPCR links functional genes with phylogenetic markers. ISME J..

[8] Hultman J., Tamminen M., Pärnänen K., Cairns J., Karkman A., Virta M.P.J. (2018). Host range of antibiotic resistance genes in wastewater treatment plant influent and effluent. FEMS Microbiol. Ecol..

[9] Roman V.L., Merlin C., Baron S., Larvor E., Ledevendec L., Virta M.P.J., Bellanger X. (2021). Abundance and environmental host range of the SXT/R391 ICE family of in aquatic microbial communities. Environ. Pollut..

[10] Bioteau A., Durand R., Burrus V. (2018). Redefinition and unification of the SXT/R391 family of integrative and conjugative elements. Appl. Environ. Microbiol..

[11] Espéli O., Moulin L., Boccard F. (2001). Transcription attenuation associated with bacterial repetitive extragenic BIME elements. J. Mol. Biol..

[12] Hochhut B., Waldor M.K. (1999). Site-specific integration of the conjugal *Vibrio cholerae* SXT element into *prfC*. Mol. Microbiol..

[13] Federation W.E., APH Association (2005). Standard Methods for the Examination of Water and Wastewater.

[14] Kirschner A.K.T., Pleininger S., Jakwerth S., Rehak S., Farnleitner A.H., Huhulescu S., Indra A. (2018). Application of three different methods to determine the prevalence, the abundance and the environmental drivers of culturable *Vibrio cholerae* in fresh and brackish bathing waters. J. Appl. Microbiol..

[15] Boulos L., Prevost M., Barbeau B., Coallier J., Desjardins R. (1999). LIVE/DEAD^®^ BacLight™: Application of a new rapid staining method for direct enumeration of viable and total bacteria in drinking water. J. Microbiol. Methods.

[16] Zhang J., Kobert K., Flouri T., Stamatakis A. (2014). PEAR: A fast and accurate Illumina Paired-End reAd mergeR. Bioinformatics.

[17] Andrews S. (2010). FastQC: A Quality Control Tool for High Throughput Sequence Data. https://www.bioinformatics.babraham.ac.uk/projects/fastqc/.

[18] Martin M. (2011). Cutadapt removes adapter sequences from high-throughput sequencing reads. EMBnet J..

[19] Edgar R.C. (2010). Search and clustering orders of magnitude faster than BLAST. Bioinformatics.

[20] R Core Team (2017). R: A Language and Environment for Statistical Computing.

[21] Madeira F., Park Y.M., Lee J., Buso N., Gur T., Madhusoodanan N., Basutkar P., Tivey A.R.N., Potter C.S., Finn R.D. (2019). The EMBL-EBI search and sequence analysis tools APIs in 2019. Nucleic Acids Res..

[22] Price M.N., Dehal P.S., Arkin A.P. (2009). FastTree: Computing large minimum evolution trees with profiles instead of a distance matrix. Mol. Biol. Evol..

[23] Letunic I., Bork P. (2019). Interactive Tree of Life (iTOL) v4: Recent updates and new developments. Nucleic Acids Res..

[24] Duhamel S., Jacquet S. (2006). Flow cytometric analysis of bacteria- and virus-like particles in lake sediments. J. Microbiol. Methods.

[25] Patel A., Noble R.T., Steele J.A., Schwalbach M.S., Hewson I., Fuhrman J.A. (2007). Virus and prokaryote enumeration from planktonic aquatic environments by epifluorescence microscopy with SYBR Green I. Nat. Protoc..

